# Seasonal patterns in trace elements assessed in toenails

**DOI:** 10.21203/rs.3.rs-3093700/v1

**Published:** 2023-06-29

**Authors:** Kaitlyn M. Wojcik, Ann Von Holle, Katie M. O’Brien, Alexandra J. White, Margaret R. Karagas, Keith E. Levine, Brian P. Jackson, Clarice R. Weinberg

**Affiliations:** National Institute of Environmental Health Sciences; National Institute of Environmental Health Sciences; National Institute of Environmental Health Sciences; National Institute of Environmental Health Sciences; Dartmouth College; Research Triangle Institute: RTI International; Dartmouth College; National Institute of Environmental Health Sciences

**Keywords:** seasonal patterns, toenail biomarkers, toenail element levels

## Abstract

Seasonal patterns in measured exposure biomarkers can cause measurement error in epidemiological studies. There is little known about the seasonality of trace elements when measured in toenails. Adjusting for such patterns when estimating associations between long-term exposures and health outcomes could be needed to improve precision and reduce bias. Our goal was to assess seasonal patterns in toenail measurements of trace elements. At enrollment, Sister Study participants, who were US residents, removed polish and collected toenail clippings, which were cleaned before analysis. We measured: iron, vanadium, aluminum, chromium, manganese, cobalt, nickel, copper, zinc, arsenic, selenium, molybdenum, cadmium, tin, antimony, mercury, and lead. For a sample of the cohort we fit trigonometric regression models with toenail element measures as the outcome, using sine and cosine functions of the collection day of the year (transformed to an angle) to assess seasonality. Results were replicated in a second sample of women, with measurements done in a separate lab. There was a seasonal association between day of collection and toenail measures for iron, aluminum, vanadium, chromium, manganese, cobalt, arsenic, molybdenum, cadmium, tin, and lead, all of which peaked near mid-August. Seasonal patterns were concordant across the two samples of women.

Given the evidence supporting seasonal patterns for 11 of the 17 elements measured in toenails, correcting for seasonality of toenail levels of those trace elements in models estimating the association between those exposures and health outcomes is important. The basis for higher concentrations in toenails collected during the summer remains unknown.

## INTRODUCTION

Measurement error in biomarkers can cause bias. Such errors can arise from issues in sample collection, processing, and storage, laboratory errors, and variability in biomarker levels over time of day or season (Tworoger 2006). When seasonal variability in biomarker levels occurs, a single measurement may not adequately represent long-term exposure. This variability is often ignored but may lead to error without careful adjustment for it (White 2011).

Levels of trace elements in toenails can be useful biomarkers of those exposures (O’Brien 2019), which could be associated with adverse health outcomes (Gutiérrez-González 2019). Toenail measures of trace elements are advantageous because nail samples are painless to collect, easy to store, and can represent long-term exposure (He 2011; Garland 1994; Were 2009). In previous studies, toenail levels for selenium, lead, manganese, cadmium, mercury, and arsenic have been found to adequately represent long-term exposure (Krogh 2003; Wu 2018; Signes-Pastor 2021). Given the slow growth rate of toenails, samples are presumed to represent exposures that occurred during the 3–12 months prior to collection (Gutiérrez-González 2019; Yaemsiri 2010).

Seasonal variation in nail biomarkers of trace element exposures could be an important source of measurement error. Seasonal patterns in trace elements measured in toenails may occur through seasonal variation in dietary intake for selenium, zinc, manganese, and arsenic (Gutiérrez-González 2019; Satia 2006; Hunter 1990; Laohaudomchok 2011; Gonzalez 2008; Slotnick 2008). Similarly, some metals, such as lead, typically have higher environmental levels in warmer weather (Levin 2020). Trace element levels in the body measured in toenails may also show seasonal patterns; however, there is little research about seasonal patterns in this type of biomarker. If seasonal patterns exist, then time of collection will contribute noise to the exposure assessment for analyses estimating associations between biomarkers and health outcomes, and the true relationship between long-term exposures to trace elements and disease risk may be obscured (Zhang 2011; Gail 2016).

Vitamin D is one example of an exposure that varies by season, with biomarker measures reflecting this seasonal pattern (Gail 2016; Bolland 2007; Wang 2009), and requiring methods to ensure these patterns do not bias estimates of associations between long-term vitamin D and disease (Wang 2009). Evidence of seasonal patterns for trace elements measured in toenails would support the importance of recording the date of sample collection and adjusting for that before modeling associations between trace metals and health outcomes. The timing and degree to which seasonal patterns exist in toenail levels of trace elements are unknown. We here assess and describe those seasonal patterns.

## METHODS

### Study Sample

The Sister Study enrolled a cohort of 50,884 women (2003–2009) between the ages of 35–74 who had not ever been diagnosed with breast cancer but had at least one sister who had (Sandler 2017). Participants provided toenail clippings collected at enrollment. Measurements were made independently by two labs based on two minimally overlapping sets of samples (O’Brien 2019; O’Brien 2019; Niehoff 2021). Our “primary” sample (n=2,900, including clippings from all toes) (Niehoff 2021) was based on women in a case-cohort study, and the “replication” sample (n=1,344; big toenails only) (O’Brien 2019) was based on women in the Two Sister Study, a sister-based study of young-onset (< age 50) breast cancer (Figure S1). There were 94 individuals who were in both samples, based on toenails they collected at enrollment, and we used those for paired analyses and then randomly assigned half of them (n=47) to toenails from one sample and half to toenails from the other sample (n=47). We had a final sample size of 2,886 people for the primary sample and 1,381 for the replication sample. We used the calculated batch adjusted values from both subsets of samples. All element values above the level of detection were used in our analyses. In the primary sample, samples that had values below the limit of detection or negative values were assigned to the limit of detection/2 (Niehoff 2021). In the replication sample, very few of the samples had batch-corrected element concentrations less than or equal to 0 μg/g; those values were reassigned to 0.001 μg/g. These element concentrations were then all log transformed for analysis.

Toenail clippings were self-collected by participants as previously described, with big toenails stored separately from the others (Sandler 2017). Participants were instructed to remove nail polish before clipping. Toenail clippings were later cleaned again in the lab and analyzed by mass spectrometry (O’Brien 2019; O’Brien 2019; Niehoff 2021.

The toenail trace elements that were measured in both the primary and replication samples from all toes included iron, chromium, manganese, nickel, cobalt, copper, zinc, arsenic, selenium, molybdenum, cadmium, tin, antimony, and lead. Aluminum measures were only assessed in the primary sample, and vanadium and mercury were only assessed in the replication sample.

All participants provided written informed consent. The institutional review board of the National Institutes of Health provided study approval and oversight.

### Statistical Methods

Descriptive statistics for the primary and replication samples included mean and standard deviation for age and median and interquartile range for body mass index. Frequencies and percentages were calculated for the three categorical variables: race/ethnicity, attained education, and smoking status. The primary sample was limited to non-Hispanic Black and non-Hispanic white women; additional races/ethnicities were included in the replication sample.

We used trigonometric regression to assess seasonal variation in toenail trace elements (Bhaskaran 2013; Stolwijk 1999; Barnett 2010). Toenail measures were fit as an outcome in a simple linear regression model. Both sine and cosine of time of year (measured in radians, as day of year, numbered from 1 to 365 multiplied by 2π/365) were included as covariates in the model. This shifted sine curve approximates a seasonal pattern.


$$\text{log}(nailmeasure)=\beta_{0}+\beta_{1}\text{sin}\left(\frac{2\pi day}{365}\right)+\beta_{2}\text{cos}\left(\frac{2\pi day}{365}\right)+e$$

We used F-tests at an alpha level of 0.05 to evaluate evidence for a seasonal effect by comparing the seasonal model with an intercept-only, time-invariant model. These models can capture peaks in trace element measures during certain months and provide estimates of relative amplitude. To estimate the timing and amplitude of peaks, we used the coefficients from the sine and cosine terms of the seasonal pattern model above to calculate the phase, which determines the time at the peak. The amplitude of the variation is the square root of the sum of $b_{1}$ squared and $b_{2}$ squared. Additionally, we compared results from the two laboratories using a Spearman correlation and by assessing the mean difference between the paired observations (Table S1). We reported the seasonal patterns for the two samples separately, as a replication.

All analyses were completed with R software, version 3.6.3 (R Core Team 2022).

## RESULTS

Descriptive results comparing the two samples are given in Table 1. Women in the primary sample (n=2,886) were older (median age of 56 years) than the women in the replication sample (n=1,381) (median age of 47 years). In terms of education levels, women in the primary sample were less likely to have a college degree than those in the replication sample (28% vs. 33%) and more likely to have a high school degree or some college but no degree (32% vs. 26%). Also, women in the primary sample were more likely to be a past smoker compared to the replication sample (36% vs 26%). More women in the primary sample were non-Hispanic Black (25%) compared to the replication sample (4.5%), a result of the primary sample only including non-Hispanic Black and non-Hispanic White women. We did not find any systematic differences in sampling by month across racial/ethnic groups. Descriptive statistics for each trace element by sample are similar (Table S2).

Starting with the primary sample, we compared models with and without terms directly modeling seasonal patterns and visually inspected violin plots (Figures S2-S3) comparing predicted monthly values to observed levels. We found evidence to support season as a predictor of toenail measures of iron, aluminum, chromium, manganese, cobalt, arsenic, molybdenum, cadmium, tin, copper, nickel, and lead. Season was not a strong predictor of toenail zinc, antimony, or selenium measures. Aluminum was measured only in the primary sample, and we found evidence to support season as a predictor (Table S3).

When there was a noteworthy seasonal pattern found in the primary sample, it agreed closely with what was seen in the replication sample. Of the two metals measured only in the replication sample, we found that time of year predicted toenail vanadium, but not mercury (Table S4). Because a log transformation was applied to all the elements, the amplitudes can be meaningfully compared among the elements and are interpretable as the estimated log of the ratio of peak to midpoint of the corresponding seasonal sine curve.

Based on the analysis of the 94 women with toenail samples sent to both labs (big toenails to one and all small toenails to the other), cadmium (r=0.77,p<.05), chromium (r=0.61,p<.05), and selenium (r=0.60,p<0.05) were found to be moderately correlated. and there was systematic disagreement between the paired measures from the same person (big toenails sent to the replication-sample lab versus all toenails sent to the primary-sample lab), with all three of the measurements being significantly lower based on the replication lab, consistent with the possibility that concentrations for those three elements may be systematically lower in clippings from big toenails. Other than those, we found little evidence that the values from the primary sample were systematically higher or lower than those from the replication sample (Table S1).

**Figure 1:**
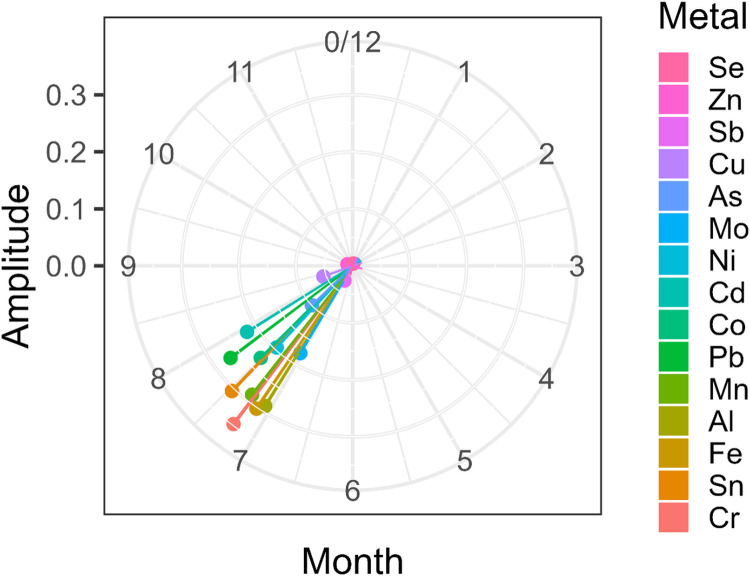
Polar plot for primary sample, showing fitted amplitude (as length) and peak day (as angle). Note that angles directed between 7 and 8 correspond to August dates.

**Figure 2:**
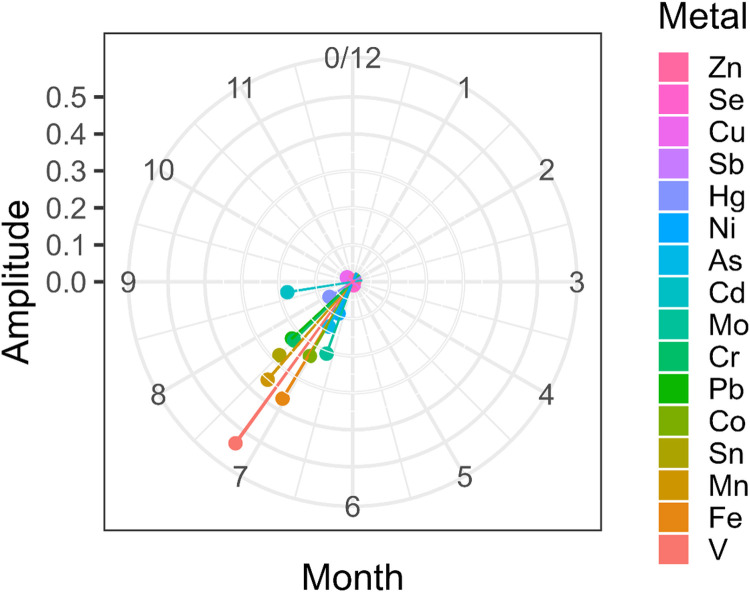
As in Figure 1, but showing fits for the replication sample.

The trigonometric models suggest that the levels for many of the studied trace elements peak in the summer months (mainly August) with the lowest levels occurring in the winter months (Figures S2-S3). See peak days listed in Table 2. These results suggest that iron, vanadium, aluminum, chromium, manganese, cobalt, arsenic, molybdenum, cadmium, tin, and lead levels in toenails are higher in summer than in winter. Chromium, iron, and tin were among the trace metals with the largest amplitude in the primary sample (Fig. 1, Table 2). Of those three, iron and manganese also had high amplitude in the replication sample (Fig. 2, Table 2). Selenium, zinc, copper, and antimony were the trace metals with the lowest amplitude in both samples.

## DISCUSSION

We used trigonometric regression models to evaluate evidence for seasonal patterns in toenail trace element measures. Seasonal patterns were apparent for 11 of the 17 elements: iron, vanadium, aluminum, chromium, manganese, cobalt, arsenic, molybdenum, cadmium, tin, and lead. Those seasonal patterns were remarkably consistent in showing a peak in August, and this pattern was repeated in the replication sample, usually with the peaks falling within two weeks of each other. These findings suggest that considering the date of clipping is important when investigating the association between trace element exposures using toenails and health outcomes, to improve the assessment of those exposures as representing long-term levels. There are a variety of methods to remove seasonal variation in biomarkers of measures such as vitamin D and phenols, which are well documented and commonly based on regression residuals (Zhang 2011; Mortamis 2012).

Several mechanisms may explain the consistent summer-peaking seasonal patterns that we found. One mechanism may include footwear differences across seasons, including open-toed shoe wear, with use being more prevalent during the warmer summer months. With open-toed shoes or bare feet, toenails are more likely to be exposed to dust/dirt with trace amounts of metals (Han 2017), potentially explaining the summer increases in some of the levels if the elements became directly embedded in the nail matrix and not incorporated during their growth. Seasonal variation in nail growth could contribute to seasonal variation in concentration. However, some of the elements showed virtually no relation to season, and one report found no evidence supporting associations between fingernail growth and seasonal patterns (Bean 1980).

Our findings raise the possibility that exposures more likely to occur during the summer months may contaminate toenail clippings and cause some of the strong seasonality we see. Although the labs carefully cleaned the nails prior to assay, many women do use nail polish on their toenails, especially in the summer, and magnesium, zinc, barium, manganese, and iron were found in a sample of 40 nail polishes (Ceballos 2021). Supporting that as a source, results in both of our samples indicated iron and manganese as having the relatively largest summer peaks (Figs. 1–2, Table 2). Interestingly, zinc, with high levels in some nail polish, does not demonstrate any seasonal patterns in either of our sets. (We note, however, that some nail clippers contain zinc, which can add noise to that measurement.) Barium and magnesium were not measured in our study. If nail polish is not fully removed before analysis or can become directly embedded in the mature nail matrix (and not from body levels when the nail first formed) and impervious to the cleaning process used in the laboratory analysis, it could cause an overestimation of metal levels in toenails (Favaro 2005). Although participants were instructed to remove nail polish before clipping their nails and the toenail clippings were cleaned thoroughly in the laboratory before the analysis, some contamination may remain, which is a potential limitation of our study, but also of wider use of toenails for assessing trace metals. Participants were not asked whether they had nail polish on their toenails just before collection, which should be done in future work.

Seasonal differences in diet may be another mechanism by which toenail trace element levels are increased in the summer. Fruit and vegetable consumption is highest in the summer and fall months, and some fruits and vegetables do contain some amounts of metals (Locke 2009; Shaheen 2016). However, toenail growth is variable, and it takes about 8–14 months to grow from base to tip. Given that there is a substantial lag relative to the time of collection, dietary exposure as a mechanism for the measured increase in toenail metals during the summer could not fully explain the observed patterns (Favaro 2005; Locke 2009). Instead, more recent exposures to the tip of the nails before collection seems more plausible. Toenail trace element levels may also vary by geographic region. Metal levels are typically higher in urban environments than in rural environments (Dhaliwal 2021). Highly polluted areas also increase the levels of metals in toenails, which may contribute to the variation in metal levels seen but not necessarily to the seasonality (Trottier 2021; Hinwood 2003; Karatela 2018).

Determining the environmental drivers of the observed summer peaks would be illuminating. For example, future research may assess the potential for nail polish to become embedded in the nail matrix that cannot be cleaned using laboratory cleaning methods. Additionally, future studies may assess the differences in seasonal patterns in warm versus cold climates. We would also like to know the role of other potential external exposures in the environment such as dust in seasonal patterns in toenail metal measures as well as fluctuations in dietary patterns.

Our estimates of seasonal patterns in many toenail trace elements document peak during the summer months. Regardless of their drivers, these patterns contribute to measurement error in epidemiological studies and adjusting for effects of the day of year of when the toenails were clipped will be necessary to improve precision and reduce bias in epidemiological studies of chronic exposures to trace elements based on toenails.

## Figures and Tables

**Table 1:** Sample characteristics Sample characteristics

Characteristic	Replication, N = 1,381 Median (IQR); n (%)	Primary, N = 2,886 Median (IQR); n (%)
**Age (years)**	47 (43, 51)	56 (50, 63)
**Education**		
Completed high school, GED, or less	149 (11%)	380 (13%)
Some college but no degree	214 (15%)	541 (19%)
Associate or technical degree	213 (15%)	419 (15%)
Bachelor’s Degree	458 (33%)	797 (28%)
Master’s Degree or other graduate level	347 (25%)	749 (26%)
**Race/Ethnicity**		
Non-Hisp White	1,234 (89%)	2,169 (75%)
Non-Hisp Black	62 (4.5%)	717 (25%)
Hispanic	46 (3.3%)	0 (0%)
Other	38 (2.8%)	0 (0%)
Unknown	1	0
**Smoking status**		
Never smoked	915 (66%)	1,592 (55%)
Past smoker	358 (26%)	1,046 (36%)
Current smoker	108 (7.8%)	247 (8.6%)
Unknown	0	1

**Table 2:** Time at peak and amplitude by type of metal and sample

	Primary	Secondary	Primary	Secondary
Metals	Amplitude	Amplitude	Time (month/day)	Time (month/day)
Chromium (Cr)	0.35 (0.32, 0.38)	0.22 (0.15, 0.35)	(08/06) 218 (212, 225)	(08/15) 226 (188, 250)
Iron (Fe)	0.30 (0.29, 0.32)	0.35 (0.33, 0.39)	(07/30) 211 (206, 217)	(07/29) 210 (205, 215)
Aluminum (Al)	0.29 (0.27, 0.33)		(07/31) 211 (206, 218)	
Tin (Sn)	0.29 (0.25, 0.34)	0.30 (0.24, 0.36)	(08/13) 224 (218, 233)	(08/15) 227 (215, 233)
Manganese (Mn)	0.29 (0.23, 0.32)	0.37 (0.34, 0.40)	(08/07) 219 (214, 223)	(08/13) 225 (215, 235)
Lead (Pb)	0.25 (0.20, 0.30)	0.22 (0.16, 0.27)	(08/22) 233 (225, 241)	(08/18) 229 (217, 254)
Cobalt (Co)	0.23 (0.19, 0.27)	0.25 (0.22, 0.29)	(08/11) 223 (218, 236)	(07/31) 211 (200, 230)
Nickel (Ni)	0.22 (0.15, 0.27)	0.13 (0.02, 0.24)	(08/09) 221 (202, 242)	(08/04) 216 (156, 267)
Cadmium (Cd)	0.21 (0.16, 0.25)	0.18 (0.14, 0.22)	(08/31) 242 (233, 256)	(09/20) 262 (251,274)
Molybdenum (Mo)	0.17 (0.14, 0.20)	0.21 (0.19, 0.23)	(07/30) 210 (198, 227)	(07/22) 202 (187, 221)
Arsenic (As)	0.10 (0.08, 0.12)	0.15 (0.11, 0.21)	(08/16) 228 (212, 238)	(07/28) 209 (189, 220)
Copper (Cu)	0.06 (0.04, 0.08)	0.02 (0.00, 0.05)	(09/07) 249 (233, 271)	(10/11) 284 (244, 347)
Antimony (Sb)	0.04 (0.01, 0.10)	0.06 (0.04, 0.10)	(08/13) 225 (85, 311)	(08/14) 226 (13, 313)
Zinc (Zn)	0.01 (0.00, 0.02)	0.01 (0.00, 0.01)	(10/11)	(06/19)
			284 (233, 348)	170 (15, 355)
Selenium (Se)	0.01 (0.00, 0.02)	0.01 (0.01, 0.02)	(06/21)	(06/13)
			171 (5, 362)	164 (114, 196)
Vanadium (V)		0.55 (0.48, 0.61)		(08/06) 218 (208, 227)
Mercury (Hg)		0.08 (0.02, 0.16)		(09/20) 263 (153, 307)

## Data Availability

The datasets analyzed during the current study are available for approved proposals through SisterStudystars.org.
